# Different degree of paternal mtDNA leakage between male and female progeny in interspecific Drosophila crosses

**DOI:** 10.1002/ece3.1069

**Published:** 2014-06-19

**Authors:** Emmanouil Dokianakis, Emmanuel D Ladoukakis

**Affiliations:** Department of Biology, University of Crete70013, Iraklion, Crete, Greece

**Keywords:** Drosophila, leakage of paternal mtDNA, mtDNA inheritance

## Abstract

Maternal transmission of mitochondrial DNA (mtDNA) in animals is thought to prevent the spread of selfish deleterious mtDNA mutations in the population. Various mechanisms have been evolved independently to prevent the entry of sperm mitochondria in the embryo. However, the increasing number of instances of paternal mtDNA leakage suggests that these mechanisms are not very effective. The destruction of sperm mitochondria in mammalian embryos is mediated by nuclear factors. Also, the destruction of paternal mitochondria in intraspecific crosses is more effective than in interspecific ones. These observations have led to the hypothesis that leakage of paternal mtDNA (and consequently mtDNA recombination owing to ensuing heteroplasmy) might be more common in inter- than in intraspecific crosses and that it should increase with phylogenetic distance of hybridizing species. We checked paternal leakage in inter- and intraspecific crosses in Drosophila and found little evidence for this hypothesis. In addition, we have observed a higher level of leakage among male than among female progeny from the same cross. This is the first report of sex-specific leakage of paternal mtDNA. It suggests that paternal mtDNA leakage might not be a stochastic result of an error-prone mechanism, but rather, it may be under complex genetic control.

## Introduction

Uniparental transmission of cytoplasmic genetic elements (i.e., mitochondria and chloroplasts) seems to be a general rule in biology. Particularly in animals, mitochondrial DNA (mtDNA) is maternally transmitted. Several mechanisms of prevention of paternal mtDNA transmission have been evolved, such as sperm with no mitochondria, destruction of sperm mitochondria in the newly fertilized egg, and eventual elimination of sperm mitochondria in the embryo by other means (Birky 2001). The variety of the mechanisms that protect uniparental transmission of mtDNA implies that these mechanisms have been evolved independently. It also suggests that there is strong selective pressure for uniparental transmission of mtDNA. The prevailing theory suggests that uniparental transmission prevents the spread of selfish deleterious mutations of cytoplasmic genetic elements in the population (Hastings 1992; Hurst 1992, 1995). This hypothesis is supported by data from several organisms such as yeast (Williamson 2002), *Neurospora* (Bertrand et al. 1980), and *C. elegans* (Clark et al. 2012). These studies have shown that mtDNA with large deletions may proliferate faster and outnumber deletion-free mtDNAs. Thus, smaller mtDNA molecules increase their representation in the mtDNA pool within the organism even though they reduce the organism's fitness. However, the validity of this hypothesis has been questioned for Drosophila (Rand 2011).

The advantage of uniparental transmission through the prevention of the spread of deleterious mutations in the population may come at the expense of deleterious mutation accumulation in the mtDNA molecule. This is because uniparental inheritance creates asexual nonrecombining lineages, which accumulate deleterious mutations faster than their sexual counterparts, a mechanism known as Muller's ratchet (Muller 1964; Felsenstein 1974; Gordo and Charlesworth 2000). Several mechanisms have been proposed to explain how mtDNA may overcome Muller's ratchet. These mechanisms include genetic bottleneck, compensatory mutations, back mutations, recruitment of mtDNA copies from the nucleus and recombination [for a review see Loewe (2006)] as well as purifying selection (Stewart et al. 2008). However, apart from recombination, the efficacy of most of these mechanisms on the elimination of Muller's ratchet has not been tested yet either on theoretical or on experimental basis. Recombination remains the main mechanism for elimination of Muller's ratchet.

Infallible uniparental inheritance of mtDNA would in effect eliminate this route of prevention of Muller's ratchet. This is because uniparental inheritance leads to homoplasmy for the maternal mtDNA (heteroplasmy due to point mutations among the mtDNA molecule of the unfertilized egg or due to mutations occurring during the life of the organism is negligible compared with heteroplasmy that could result from biparental inheritance). In a population of identical mtDNA molecules, recombination generates molecules that are identical to themselves and to parental molecules. Thus, prevention of mutation accumulation and eventual mutation accumulation of the mtDNA molecule would be inevitable. Might, then, leakage of paternal mtDNA have evolved because it provided a means for overcoming this limitation?

Despite the wealth of mechanisms promoting uniparental transmission of mtDNA, paternal mtDNA has been observed occasionally in several animal species such as mouse (Gyllensten et al. 1991), Drosophila (Kondo et al. 1992; Sherengul et al. 2006; Nunes et al. 2013), anchovy (Magoulas and Zouros 1993), sheep (Zhao et al. 2004), and human (Schwartz and Vissing 2002). In all these cases, paternal mtDNA is a small minority in the embryo compared with the maternal mtDNA. Leakage of paternal mtDNA in the embryo has been explained as a breakdown of the mechanisms that promote uniparental transmission. Kaneda et al. (1995) observed that paternal mitochondria are eliminated in intra- but not in interspecific crosses in mouse. Sperm mitochondria in mammals are tagged with ubiquitin during spermatogenesis leading to their recognition and subsequent degradation by the proteolytic machinery in eggs after fertilization (Sutovsky et al. 1999, 2000). Based on these observations, Rokas et al. (2003) proposed a conceptual model for paternal mtDNA leakage which allows mtDNA recombination to occur. According to this model, elimination of sperm mtDNA in the fertilized egg involves a reaction between a nuclearly encoded factor that labels the sperm mitochondria and an also nuclearly encoded factor in the egg cytoplasm. The recognition of the label of sperm's mitochondria by the egg factor is nearly perfect in homospecific crosses because they are both encoded by the same nuclear background. In heterospecific crosses, however, the egg factor and the label of sperm mitochondria are encoded from different nuclear backgrounds and the recognition of each other might be less effective allowing the maintenance of some paternal mitochondria in the egg. The recognition of the sperm mitochondria by the egg factor would become less effective, the more distant are the species involved in the cross because more nonshared mutations would have accumulated in the two independently evolving taxa. The model makes two testable predictions. The first prediction is qualitative and suggests that the leakage of paternal mtDNA into the embryo would be more common in interspecific than in intraspecific crosses. The second prediction is quantitative and suggests that the level of leakage would increase as the genetic distance between the hybridizing species increases. In this study, we test these two hypotheses using Drosophila inter- and intraspecific crosses. If the model is correct, we expect that leakage of paternal mtDNA would be higher in inter- than in intraspecific crosses and that we would find more hybrids with leakage in crosses between more divergent Drosophila species. Furthermore, we detected the presence of paternal mtDNA in individual hybrids. The differential presence of paternal mtDNA in male and female hybrids would be good indication that leakage of paternal mtDNA is not a random phenomenon but might be under genetic control.

## Materials and Methods

We used the following species of Drosophila: *D. simulans, D. melanogaster* (Oregon R)*, D. teissieri, D. sechellia, D. mauritiana, D. yakuba,* and *D. santomea*. Populations of *D. simulans* carry three and *D. mauritiana* carry two distinct mitochondrial haplotypes (mitotypes) depending on their geographic origin (Ballard 2000a,b). We used two *D. simulans* mitotypes (*si*I and *si*II) and one *D. mauritiana* mitotype (*ma*II). Most of the strains we used have been kindly provided by Prof. K. Bourtzis (University of Ioannina, Greece) from the stocks published in Bourtzis et al. (1996). Also Drs F. Missirlis (Queen's Mary University, UK) and I. Ibba (Max Planck Institute for Chemical Ecology, Germany) provided *D. sechellia* and *D. simulans*. Flies were grown at 25°C ± 1°C with 12-h photoperiod on standard medium (Ashburner and Scott Hawley 2004). Both inter- and intraspecific crosses were set up in plastic vials, containing standard medium, with three virgin females and nine males. Practice suggests an excess of males in interspecific crosses (Hammerle and Ferrus 2003; Sherengul et al. 2006; Gerard and Presgraves 2012). Parents were removed when pupae were visible. When adult offspring emerged, their sex was determined under a stereoscope and stored in −80°C until DNA extraction (see below). For each cross, we set up three replicate vials. All crosses we performed are shown in Table 1. In the notation of the crosses, the first species is always the maternal species.

**Table 1:** Crosses and leakage of paternal mtDNA. Column 4 gives the divergence between parental species based on the *adh* locus. The last three columns give the number (and percentage) of progeny that tested positive for paternal mtDNA

Female parent	Male parent	Divergence	Repeats	No of male/female offspring	Male offspring (%)	Female offspring (%)	Male and female offspring combined (%)
1. *D. yakuba*	*D. mauritiana*	0.076	4	5/5	5/5 (100)	4/5 (80)	9/10 (90)
2. *D. teissieri*	*D. mauritiana*	0.070	2	10/25	10/10 (100)	6/25 (24)	16/35 (46)
3. *D. melanogaster*	*D. mauritiana*	0.031	1	0/30	0/0 (0)	0/30 (0)	0/30 (0)
4. *D. santomea*	*D. mauritiana*	0.077	1	0/50	0/0 (0)	0/50 (0)	0/50 (0)
5. *D. simulans*	*D. mauritiana*	0.019	1	40/30	40/40 (100)	3/30 (10)	43/70 (61)
6. *D. sechellia*	*D. mauritiana*	0.015	2	No offspring			
7. *D. simulans*	*D. melanogaster*	0.021	2	No offspring			
8. *D. mauritiana*	*D. melanogaster*	0.031	3	16/1	0/16 (0)	0/1 (0)	0/17 (0)
9. *D. sechellia*	*D. melanogaster*	0.024	2	No offspring			
10. *D. melanogaster*	*D. sechellia*	0.024	2	0/28	0/0 (0)	0/28 (0)	0/28 (0)
11. *D. simulans*	*D. sechellia*	0.009	1	30/20	28/30 (93)	0/20 (0)	28/50 (56)
12. *D. mauritiana*	*D. sechellia*	0.015	2	No offspring			
13. *D. melanogaster*	*D. simulans*	0.021	4	No offspring			
14. *D. mauritiana*	*D. simulans*	0.019	4	13/15	0/13 (0)	0/15 (0)	0/28 (0)
15. *D. simulans (I)*	*D. simulans (II)*	0	3	100+	0/20 (0)	0/20 (0)	0/40 (0)
16. *D. simulans (II)*	*D. simulans (I)*	0	1	100+	0/30 (0)	0/20 (0)	0/50 (0)

Primers specificity was tested on DNA extracted from pools of 6-10 flies taken directly from the cultures, according to Miller et al. (1988). DNA concentration was assessed using a Nanodrop spectrophotometer (Nanodrop Technologies, Oxfordshire, UK).

We applied single-fly DNA extraction to detect presence of paternal mtDNA. Individual flies were homogenized in 49 μL freshly prepared extraction buffer (1 mmol/L EDTA, 25 mmol/L NaCl, 10 mmol/L Tris-HCl, pH 8.0). 1 μL proteinase K was added from a stock 10 mg/mL. The mixture was incubated at 37°C for half an hour. The enzyme was deactivated by incubating the mixture at 95°C for 5 min. After cooling down to room temperature, 1 μL from the mixture was used as template for PCR.

Some of the primers used in this study were already published (Table 2). Species-specific primers were designed by comparing fully sequenced mtDNAs from *Drosophila* species after aligning them with ClustalW (Thompson et al. 2002) as implemented in MEGA5 package (Tamura et al. 2011).

**Table 2:** Primers used for each cross of this study and substrate dilution limits for paternal mtDNA detection. All pairs of primers apart from 12SAIF/12SBIR have been used to detect paternal mtDNA of crosses from Table 1

Primer name	Sequence	Annealing temperature (°C)	Substrate dilution limit	Cross numbers (from Table 1)	Reference
12SAIF	AAACTAGGATTAGATACCCTATTAT	59	–	All	Simon et al. (1994)
12SBIR	AAGAGCGACGGGCGATGTGT				
mauyakF	ATATTATTCGACCTGGAACA	59	10^−7^	1 & 2	Present study
mauyakR	CTCCTAATTCAATAGCGGGT				
maumelF	TTACTCCTTCAAAATTGCAGTTTGAT	59	10^−6^	3	Present study
maumelR	CCTGCTAATACTGGTAATGATAAA				
mausanF	GCTATAGCCGCTGGTAACCA	63	10^−5^	4	Present study
mausanR	TATGGCAGCTCCTCCTACAT				
mausimF	GCTATTGGAGGTTTAAATCAG	56	10^−5^	5	Present study
mausimR	AATTCTTAGGGATGTACCT				
melOR_1594F	GCTGAATTAGGACATCCTGGAGC	58	10^−3^	8	Present study
melOR_2385R	TCGAGTATCTACATCTATTCCAACG				
sech_6676F	TAATTGACCGTAATTCAATGGG	58	10^−4^	10 & 11	Present study
sech_7614R	GCAGCTATGGCTGCCCCTACT				
simII_5183F	TTCAGGAGTTACTGTAACC	58	10^−4^	14 & 15	Dean et al. (2003)
sim_uni_5983R	TATTCCTTGATTTCATTCATG				
simI_1737F	TCCTGATATAGCATTTCCA	57	10^−4^	16	Present study
simI_2531R	GTTAATCCTCCTACTGTG				

All PCR assays were carried out in a Biometra T-personal Thermocycler (Biometra biomedizinische Analytik GmbH, Germany). To achieve the best specificity for each primer pair, we tried several PCR conditions of annealing temperature, concentration of MgCl_2_ (1.5 mmol/L, 2.5 mmol/L and 3 mmol/L), and time intervals of each PCR step. We arrived at the following conditions: 94°C for 5 min followed by 42 cycles of 94°C for 30 s, annealing temperature according to each pair of primers (see Table 2) for 30 s and 72°C for 30 s, with a final extension step of 10 min at 72°C. Each PCR (15 μL) contained 1X *Taq* polymerase buffer (Minotech Biotechnology, IMBB, Greece), 2.5 mmol/L MgCl_2_, 0.3 mmol/L of each primer, 1 μL template DNA, 0.3 mmol/L of each dNTP, and 0.5 units *Taq* DNA polymerase (Minotech Biotechnology, IMBB, Greece).

To find the limit of the detection of template DNA for each pair of primers, we started from an initial concentration of approximately 1 μg/μL of total DNA and made a series of dilutions from 1:1 down to 1:10^−9^. We performed the PCR using the optimal conditions of each pair of primers. The limit of detection for each pair of primers was defined as the last concentration for which we obtained a visible PCR product.

To test for the presence of paternal mtDNA in an individual fly, we performed the following procedure: First, we tested the quality of mtDNA of the fly with a PCR assay using 12S RNA primers (Simon et al. 1994). Individuals that failed to produce amplicons in this step were excluded from the subsequent steps of the analysis. Second, for individuals that were successful in the first step, we performed a second PCR assay targeting the paternal mtDNA and using the appropriate primer sets from Table 2. We considered individuals that were positive to this assay as having paternal mtDNA.

Although the protocol for detecting leakage of paternal mtDNA using PCR considered reliable, the ultimate confirmation for leakage of paternal mtDNA was to compare the sequence of the PCR product from hybrids with that of the paternal species of hybrids. The amplicons were purified using NucleoSpin Extract II kit (Macherey – Nagel, Germany) according to manufacturer's instructions. DNA from these bands was quantified in a Nanodrop spectrophotometer (Nanodrop Technologies, Oxfordshire, UK), and five samples from each cross were sequenced to confirm that they belonged to paternal mtDNA (see Results section). For completeness, we also sequenced PCR products from the paternal species.

In addition to sequencing the mtDNA of all seven species examined, we used a mitochondrial (*nad5*) and two nuclear (*adh* and *period*) genes for phylogenetic analysis. The accession numbers for *adh* sequences were X54118, X54120, X00607, M19264, X04672, Z00030, AY804554; for *period* sequences, AF251251.1 AF251254, AF251250, AF251240, AF251249, AF251241, AF251248; and for *nad5* sequences, U37541, DQ383113, DQ383070, NC001322, AF200832, AF200830, AF200838. Sequences were aligned with ClustalW (Thompson et al. 2002), and genetic distances among sequences were estimated with MEGA5 using the model maximum composite likelihood (Tamura et al. 2011).

Statistical analysis of the results was performed using SPSS version 19 for Mac OS X (IMB SPSS statistics), and Excel (Microsoft). This analysis included the chi-square test and the Spearman correlation test between leakage and divergence.

## Results

### Assessing primer specificity and detection limits

We tested the specificity of primers and the optimal PCR conditions for paternal mtDNA detection in each cross. For example, in the cross *D*. *teissieri* x *D*. *mauritiana,* we tested whether the primers amplified *D*. *mauritiana*'s mtDNA (which is the paternal mtDNA) but not *D. teissieri*'s mtDNA. We aimed at obtaining the highest specificity rather than the maximum yield of PCR product.

**Figure 1:**
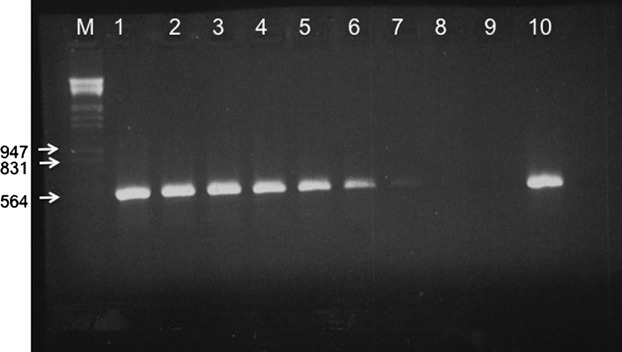
Target DNA detection limit for the primers mauyakF and mauyakR. M: marker λ/*Hind*III/*Eco*RI. Lanes 1 to 9 correspond to dilutions 1:10–1:10^−9^, 10: undiluted 1 μg total DNA from *D. mauritiana*. The expected product is 569 bp. The limit of detection is 1:10^−7^.

We performed a set of PCRs to find the limit at which the PCR could amplify the paternal mtDNA. Different pairs of primers had different limits for mtDNA detection. The lowest limit (1:10^−7^) was for primers for *D*. *mauritiana*'s mtDNA in the cross *D*. *yakuba* x *D*. *mauritiana* (Fig. 1). The highest limit (1:10^−3^) was for the primers used to detect *D*. *melanogaster*'s mtDNA (Table 2).

### Reproductive success, fecundity, and F1 sex ratio

We performed 14 interspecific crosses between species of the *D*. *melanogaster* species complex, which according to the literature could produce hybrids (Lachaise et al. 1986, 2000; Lee and Watanabe 1987). Five crosses failed to produce any progeny despite the fact that they were repeated for at least two times each (*D*. *simulans* x *D. melanogaster*, *D*. *sechellia* x *D*. *mauritiana*, *D*. *sechellia* x *D*. *melanogaster*, *D*. *mauritiana* x *D*. *sechellia,* and *D*. *melanogaster* x *D*. *simulans*). The remaining nine interspecific crosses produced variable numbers of progeny. The sex ratio differed between crosses, with some crosses revealing ratio 1:1 (e.g., *D*. *yakuba* x *D*. *mauritiana*) and others producing exclusively or almost exclusively one or the other sex (Table 1). We also performed two intraspecific crosses between two strains of *D. simulans* with different mitotypes, *si*I and *si*II, (Ballard 2000a). As expected, these intraspecific crosses were more successful than the interspecific ones and produced male and female progeny at a ratio 1:1 (Table 1).

### Leakage of paternal mtDNA

**Figure 2:**
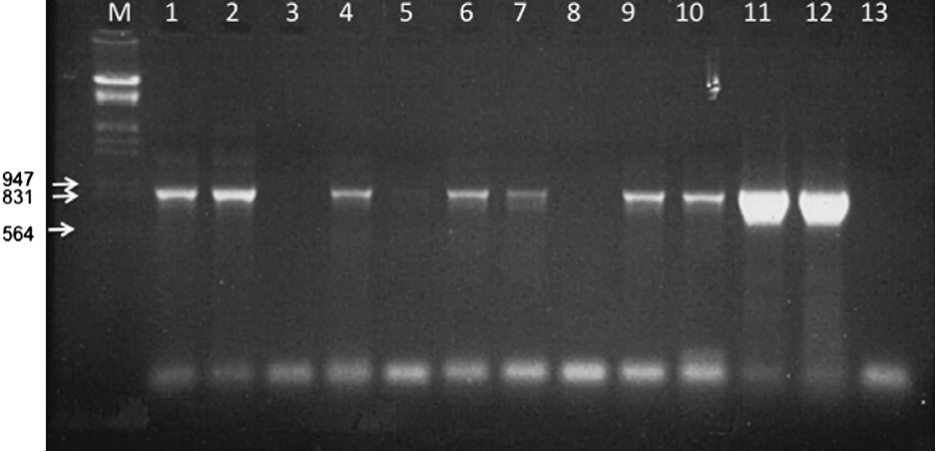
Leakage of paternal (*D. sechellia*) mtDNA in the cross *D. simulans* x *D. sechellia*. Individuals in lanes 1, 2, 4, 5, 6, 7, 9, and 10 are male hybrids. Individuals in lanes 3 and 8 are female hybrids. Lanes 11 and 12 are positive controls (*D. sechellia* total DNA). Lane 13 is negative control (*D. simulans* total DNA). The primers for the PCR were sech6676F/7614R, and the expected size of the product is 938 bp. M is the size marker λ/*Hind*III/*Eco*RI.

We counted the progeny that contained paternal mtDNA in levels higher than the detection limit of each primer pair (Fig. 2 and Table 1). Because template sensitivity differed between single pairs of primers, we first examined whether the occurrence of paternal leakage was correlated with increased template sensitivity. We found no correlation (*P* = 0.344). Thus, any difference at the proportions of hybrids with leakage among crosses cannot be attributed to differences in primers sensitivity.

We observed no leakage of paternal mtDNA in interspecific crosses that produced only female progeny (*D. melanogaster* x *D*. *mauritiana*, *D*. *santomea* x *D*. *mauritiana*, *D*. *melanogaster* x *D*. *sechellia*). Also, we observed no leakage in the cross *D. mauritiana* x *D. melanogaster* that produced almost exclusively male progeny (it produced only one female among 17 hybrids). Finally, we observed no leakage of paternal mtDNA in the cross *D*. *mauritiana* x *D*. *simulans* which produced 13 males and 15 females (sex ratio not different from 1:1).

Leakage of paternal mtDNA was observed in four interspecific crosses: *D. yakuba* x *D. mauritiana*, *D. teissieri* x *D. mauritiana*, *D. simulans* x *D. mauritiana,* and *D. simulans* x *D. sechellia*. All these crosses produced hybrids of both genders, albeit not always in 1:1 ratio. In all four crosses, leakage was a common phenomenon: In the cross *D. yakuba* x *D. mauritiana,* nine of 10 hybrids contained paternal mtDNA, in *D. teissieri* x *D. mauritiana* 16 of 35, in *D. simulans* x *D. mauritiana* 43 of 70, and in *D. simulans* x *D. sechellia* 28 of 50 (Table 1). Interestingly, in three crosses, the proportion of hybrids with paternal mtDNA was not the same between the two sexes. Specifically, leakage was significantly more common among male progeny. In *D. teissieri* x *D. mauritiana,* all males and six of 25 females contained paternal mtDNA (*P* value ≪ 2*10^−14^); in *D. simulans* x *D. mauritiana,* all males and three of 40 females contained paternal mtDNA (*P* value ≈ 0); and in *D. simulans* x *D. sechellia,* 28 of 30 males and no female contained paternal mtDNA (*P* value ≈ 0). The cross *D. yakuba* x *D. mauritiana* was an exception, with all five males and four of five females containing paternal mtDNA. The difference in leakage between sexes for this cross was not statistically different (*P* value = 0.48), but this might occur because of the overall small number of progeny. We observed no leakage of paternal mtDNA in either male or female progeny in the two reciprocal intraspecific crosses of *si*I and *si*II.

We divided the crosses into those with leakage and those without leakage. In the first class, leakage was observed in almost 100% (average 98.5%) among male progeny, and the among-males leakage was equally frequent among crosses (*P* = 0.289). Among female progeny, leakage was much lower (16.3% on average) and varied among crosses (*P* = 0.0001).

**Figure 3:**
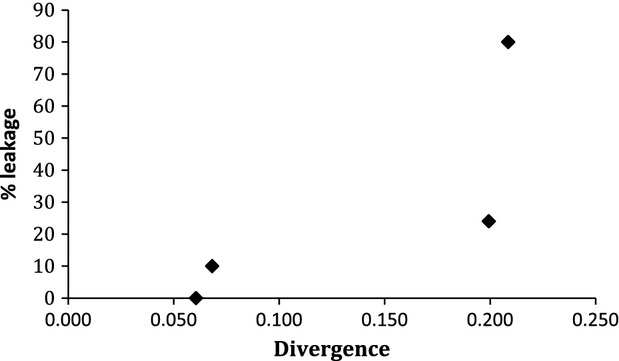
Percentage of paternal mtDNA presence in female offspring in the four crosses in which leakage of paternal mtDNA was detected. Divergence was calculated for the nuclear gene *period*. *Adh* (nuclear) and *nad5* (mitochondrial) genes produced similar pictures.

We have examined whether the occurrence of progeny that contained paternal mtDNA was correlated with the genetic distance (divergence) of the parental species. To quantify the genetic distance, we used three genes, two nuclear (*adh* and *period*) and one mitochondrial (*nad5*), for which sequences for all seven *Drosophila* species were available in GenBank. The test for a possible correlation was conducted for each of the three genes separately. We performed four Spearman correlations. First, we correlated the divergence between parental species with percentage of leakage (with no reference to the sex of progeny) for all nine successful interspecific crosses. We found no correlation (*P* = 0.832), but this result might be biased because five of the nine crosses produced progeny with no paternal mtDNA. Second, we performed the same test for only the four crosses in which we observed leakage. Again there was no correlation (*P* = 0.600). For these four crosses, we performed Spearman correlation between the divergence of the parental species and the leakage in male and female progeny, separately. These sex-specific tests produced no evidence for correlation for males (*P* = 0.255), but produced a significant correlation for females (Spearman correlation coefficient ≈1, *P* value < 0.01) (Fig. 3). These results were the same for each of the three genes (data not shown).

## Discussion

Leakage of paternal mtDNA in animal embryos occurs rarely because several mechanisms protect uniparental transmission of mtDNA (Birky 1995). However, there is an increasing list of cases where paternal mtDNA has been observed at low proportion (Magoulas and Zouros 1993; Shitara et al. 1998; Schwartz and Vissing 2002; Zeh and Zeh 2005; Sherengul et al. 2006; Nunes et al. 2013). Rokas et al. (2003) proposed a model about the degree of paternal mtDNA leakage. The model proposes that the leakage should be higher in inter- than in intraspecific crosses and that its degree should increase with the divergence of hybridizing species.

Our results agree in part with the first prediction of this model. We observed leakage in inter- but not in intraspecific crosses. Yet, leakage was observed in only four of the nine interspecific crosses. Previous studies have reported a higher incidence of leakage in interspecific than in intraspecific crosses in mice (Kaneda et al. 1995; Shitara et al. 1998) and in Drosophila (Kondo et al. 1990, 1992; Sherengul et al. 2006). The observation of no leakage in five interspecific crosses may suggest that leakage occurs at low frequency in these crosses, thus requiring larger sample sizes for its detection. It is worth noting, however, that one of the five hybrid crosses that showed no leakage is also the one with the highest divergence (Table 1, cross # 4) and that one of the four hybrid crosses that showed leakage was the one with the smallest divergence (Table 1, cross #11).

We detected no leakage of paternal mtDNA in two reciprocal intraspecific crosses of *D. simulans* (between mitotypes *si*I and *si*II). The result was the same regardless of the direction of the cross. Wolff et al. (2013) addressed the same question using crosses between two strains of *D. simulans* with different mitotypes (*si*II and *si*III). They detected leakage in less than 1% of the progeny (27 of 4092). This level of leakage could easily escape our detection that was based on the examination of less than 50 hybrids in the two intraspecific *D. simulans* crosses. In natural populations of *D. melanogaster,* 14% of the individuals were found to be heteroplasmic, suggesting that leakage might be more common in that species (Nunes et al. 2013).

Clearly, leakage does not appear to depend upon genetic distance of parental species, but it may depend on the maternal species. Within the group of the five crosses without leakage, two crosses were mothered by *D. melanogaster* and two crosses by *D. mauritiana*. In the group with leakage, two of the four crosses were mothered by *D. simulans*. *D. santomea* produced no leakage when crossed to *D. mauritiana*, but it was not used in any other cross to see if it would produce the same result. Likewise, *D. yakuba* and *D. teissieri* produced progeny with leakage when crossed to males of *D. mauritiana,* but no other crosses exist with these species as maternal species. No such trend exists for the paternal species. *D. mauritiana* fathered five hybrid crosses of which two showed no leakage and three did. Likewise, *D. sechellia* fathered two crosses, of which one showed no leakage and one did. The possibility of a maternal effect on the possibility of paternal mtDNAs leakage remains, however, ill-supported owing to the small number of crosses on which it is based.

In crosses in which paternal mtDNA leakage occurs, the number of male progeny bearing paternal mtDNA is much larger than that of female. This is an unexpected and, apparently, very interesting result. It suggests that in a hybrid cross, the mechanism(s) that prevent paternal mtDNA leakage would either break down or not, but when it does the break will affect disproportionally the two genders, with the males been the ones most affected. Indeed, it appears that in crosses in which the mechanism breaks down, practically all males will inherit paternal mtDNA, whereas the percentage of females with paternal mtDNA may depend on the genetic distance between the two parental species (Fig. 3).

These observations suggest that leakage of paternal mtDNA might not in fact be a “leakage”, in the sense that it results from the breakdown of a protective mechanism, which once it occurred it allows the paternal mtDNA to leak randomly among progeny. Rather, it may be under the control of complex machinery. This view is supported by the fact that leakage is totally absent in some crosses and present in others, irrespective of phylogenetic distance, that when leakage occurs, its rate of occurrence varies dramatically among male and female progeny and, also, from the fact that no leakage was observed in the offspring of the three crosses that produced only one sex.

Although highly speculative, one may advance the hypothesis that paternal leakage occurs regularly at low frequencies to avert mutational meltdown of the mitochondrial genome. Clonal transmission of mtDNA produces recombination-free lineages. Nonrecombining genomes suffer from the accumulation of deleterious mutations, a mechanism which is known as Muller's ratchet (Muller 1964; Felsenstein 1974). Using a realistic range of values about mutation accumulation and selection, Loewe (2006) suggested that Muller's ratchet should have led to the collapse of mtDNA. The survival of uniparentally transmitted mtDNA from the accumulation of deleterious mutation has been called “the mitochondrial DNA paradox” (Loewe 2006). Hoekstra (2000) has put forward the idea that natural selection might allow paternal mtDNA to remain in the embryo in amounts small enough that uniparental transmission would not be compromised, yet large enough for recombination to occur at a rate sufficient to counteract Muller's ratchet. Theoretical studies have shown that very little recombination (sometimes undetectable) is needed to cancel out Muller's ratchet (Gordo and Charlesworth 2001; Loewe 2006; Neiman and Taylor 2009). The first step for recombination to occur is heteroplasmy, which has been observed in several animal species (Gyllensten et al. 1991; Magoulas and Zouros 1993; Zeh and Zeh 2005; Sherengul et al. 2006; Wolff et al. 2013). Also mtDNA recombination has been observed in animals either directly or indirectly (Ladoukakis and Zouros 2001; Kraytsberg et al. 2004; Piganeau et al. 2004; Tsaousis et al. 2005; Ladoukakis et al. 2011). A well known case of human heteroplasmy (Schwartz and Vissing 2002) was found to lead to recombination (Kraytsberg et al. 2004). Therefore, both leakage of paternal mtDNA and mtDNA recombination are known to occur. Both uniparental mtDNA inheritance and avoidance of mutational meltdown of mtDNA can be achieved, provided there are mechanisms in place that ensure that leakage of paternal mtDNA does not exceed a specific threshold. The differential leakage of paternal mtDNA among male and female progeny is an indirect evidence for the existence of such mechanisms, which, however, remain to be studied in finer detail.

One could argue that the phenomenon of leakage would be more important in hybrid zones, particularly for those species that backcrossing is feasible. However, recent studies have reported leakage of paternal mtDNA in intraspecific crosses of *D. simulans* (Wolff et al. 2013) and in natural populations of *D. melanogaster* (Nunes et al. 2013). These reports suggest that the difference between interspecific and intraspecific crosses in mtDNA leakage is quantitative; there is more leakage in interspecific crosses, but leakage occurs also in intraspecific crosses. This makes the study of leakage easier in hybrid crosses, but one would hope that the results from such crosses could be extended to pure crosses. In particular, it would be of extreme interest if it can be shown that paternal mtDNA inheritance is more common among male than among female progeny in intraspecific crosses. Further studies using crosses within and between species in Drosophila and other organisms are needed to confirm that the mechanism reported in this study is general or not. The next interesting step is to recognize which is the mechanism that controls the leakage of paternal mtDNA in the embryos.
